# Factors related to healthy sexual and contraceptive behaviors in undergraduate students at university of Seville: a cross- sectional study

**DOI:** 10.1186/s12978-017-0444-9

**Published:** 2017-12-29

**Authors:** Fátima Leon-Larios, Juana Macías-Seda

**Affiliations:** Departamento de Enfermería, Facultad de Enfermería, Fisioterapia y Podología, Universidad de Sevilla, Calle Avenzoar, 6, 41009 Sevilla, Spain

## Abstract

**Background:**

Young people are a group of population with sexual risk-taking behaviors. Despite efforts to inform them it is common for them to adopt risk conducts during their stay at University. The aim of this research was to assess knowledge, attitudes and experiences on sexual health and contraceptives and factors related to risk behaviors in university students.

**Methods:**

A cross- sectional analytical study was carried out from February to April 2014 among undergraduate students at University of Seville. A self-administered questionnaire was filled in by participants. Data analysis was performed using SPSS V22. Descriptive statistics were used to show data. A *P*-value of 5% (two-tailed) was considered statistically significant.

**Results:**

A total of 566 students responded to the questionnaire. 47.3% (267) were male and 52.6% (297) female. About sexual behavior: 93.3% of participants were sexually active last year. 58.3% had had sex under alcohol effect and 18% under drugs effect. About contraceptive behavior: 86.9% used a contraceptive method during their first sexual relation, the male condom being the most used (90.6%). Currently, the most used contraceptives are the male condom, and hormonal pills. The participants’ answers about their knowledge on contraceptives and STIs (sexual transmission infections) showed weaknesses. Participants who had received sexual and contraceptive education showed more knowledge (*p* < 0.001). We did not find differences about knowledge on contraceptives and STIs by age (*p* = 0.056). Level of knowledge is less in young people who use coitus interruptus or none as a contraceptive method (*p* < 0.001). We observed differences by frequency of sex since young people who had sex more frequently showed more knowledge about contraceptives and STI (*p* < 0.001). There are more women that had a partner than men (*p* = 0.003) and their attitudes and experiences on sex are healthier. Females showed more knowledge about management of hormonal contraceptives and medical controls (*p* < 0.001).

**Conclusion:**

Factors that contribute to having a healthier behavior on sexuality and contraception are age, gender and background in health issues, showing greater knowledge and less risky behavior. Programing reproductive health programs at university should be continued.

## Plain summary

A cross- sectional study was carried out with students from different backgrounds at University of Seville (Spain) with the objective of assessing knowledge, attitudes and experiences on sexual health and contraceptive behavior. Participants were sexually active, and the male condom and contraceptive pills were the contraceptive method that was most used. They recognized having had sex under the effect of alcohol and drugs. Their knowledge about sexual transmission infections is weak and should be improved. Participants who had attended some kind of course about the topic showed a healthier sexual behavior. Gender is a factor to be taken into consideration, woman had less risky behaviors than men and had more information about sexual and medical controls. It is necessary to continue working on reproductive health programs at university with young people.

## Background

Adolescent and young people are more sexually active than any other group of the population. They show sexual risk-taking behaviors and an early age of sexual initiation, unplanned sex and several sexual couples [1]. Otherwise, they are more likely to use contraception inconsistently and this makes them a very vulnerable segment of the population [2, 3].

The non-use of contraceptives has a consequence of not only unplanned pregnancies but also sexually transmitted infections. The rates of STI among young people are more often every day. It is estimated that 15–24 years old youths account for half of all new STI infections [4]. According to WHO, each year, there are an estimated 357 million new infections with 1 out of 4 STIs: chlamydia, gonorrhea, syphilis and trichomoniasis [5], In Spain, data from different studies also reported a high prevalence of STI in the last year [6, 7].

For many years, governments and organizations have dedicated efforts on sexual and reproductive health educating young people [8]. School curriculums on sexual health at secondary education level are more common every day. Despite the fact that these programs were implemented throughout the world, they share many aspects in common [9].

Sexual and contraceptive education is a good tool to ensure that young people have the information needed to follow a healthy sexual life [10]. Schools have the potential for reducing adolescent sexual risk-taking [11]. The question of whether sexual education is being effective has been assessed in some studies [2, 12]. COMPAS and CUIDATE programs were carried out in Spain and showed effectiveness when using contraceptives and knowledge about STI, and less effectiveness in maintaining the intention to use condoms and the delay of their first oral sex experience. Intention was measured and had positive findings; otherwise, neither intervention had a long-term impact on behavioral variables [12].

Knowledge, attitudes and practices related to contraceptives and sexual behaviors are crucial to influence a healthy lifestyle [13]. Young people have to learn about sexuality, STI and contraception [3, 14]. However, studies show that receiving the information is not always related to adequate sexual and contraceptive attitude and more effective strategies of behavioral change in individuals who engage in sexual activity are needed [15, 16]. On the other hand, information is not synonymous of a healthy lifestyle. Information and motivation may influence behavioral skills which produce behavior changes [17].

Several studies focus on the importance of sexual education from the earliest school years [18]. Programs alone cannot avoid risking behaviors in young people, but may change sexual and protective behaviors in desired directions and may be an important component in larger initiatives [9].

A review performed by Kirby [9], concluded that adolescents that attended programs about sexuality, contraception and prevention of STIs more often had fewer sexual risk behaviors.

In Andalusia, the population of this study, a program that includes sexual and reproductive education was taught in secondary schools called FORMA JOVEN from 2001.

In the guideline of “Forma Joven” the schools included are required to offer 6 to 10 h of health education weekly, or even more than 15 h in some of them [19]. However, this initiative is widely considered to be inadequate for its insufficient impact on health styles, proven by the fact that no participating students obtained similar scores in studies carried out [20]. If we add lack of formal sexual education from parents, [21, 22] it turns out that young people are uninformed and need to obtain information from other sources.

Age for the first intercourse is around 14–16 years according studies published [23] so when these students are enrolled at university, they already have an active sexual life. This study aims to explore students’ knowledge, attitudes and experiences on sexual health and contraceptives and factors related to risk behaviors to develop educational programs at university, if needed.

## Methods

### Study design and setting

A cross sectional analytic study was conducted from February to April 2014 at the University of Seville. We recruited female and male undergraduates from different degrees.

Student were selected according to the year of study from all knowledge areas: Health Sciences, Social Sciences, Engineering and Architecture; Sciences and; Humanities and Art.

A total of 3.349 students were randomly selected by groups (classes). The final sample size was estimated to give the study a confident interval of 99%, distribution of answers of 50% and estimated error of 5%. Inclusion criteria was acceptance of informed consent and to be enrolled in any degree at University of Seville.

The outcomes variables included knowledge about contraception, sexual behavior and contraceptive use. Other variables were sex, age of participant, education level and religion.

### Data collection

Prior to data collection, the questionnaire was piloted among 30 students to identify possible errors. Linguistic difficulties were not found.

The test was administered to students in a class at university to answer it. This was a self-administered questionnaire with closed and open questions.

The questionnaire was structured in three parts: one, about sociodemographic characteristics. The second part, about respondent’s sexual experience and contraceptive usage. The third part, assessed knowledge on contraceptives and sexual transmission infections (STI) through 9 items developed by the authors and based on the literature documenting some frequent errors about contraceptive methods and STI. Participants had to answer to nine dichotomous items with true or false:

1. Coitus interruptus is a very safe contraceptive.
2. If a condom is washed carefully, it is possible to reuse it several times.
3. To use hormonal pills, you have to have an appointment with a general practitioner at primary health Centre.
4. Having sex during menstruation is safe to prevent pregnancies.
5. Hormonal pills are effective if you take them before sex.
6. Hormonal pills protect against STI.
7. The only method that protects you against STI is a condom.
8. All sexually active women should perform a Papanicolau annually.
9. STI are common in our society.

Every right answer was evaluated with 1 point, the minimum being 0 points and maximum 9 points.

This brief questionnaire was validated and showed internal and external consistency. Internal consistency reliability was measured with Kuder-Richardson formula 20 (KR-20) for dichotomous scores and was equal to 0,57. In addition, an exploratory factorial analysis was performed (KMO = 0,67; χ2(28, *N* = 566) =350,77; *p*<,001), and 3 factors were found with a self-value higher than 1, explaining variance of 54,79%. The model presents a good global adjustment χ2(14, *N* = 566) =17,48, *p* = .232. Quotient χ^2^ among liberty grades is inferior to a 3 (17,48/14 = 1,25), and the valor of CFI is 0,97 and RMSEA is de 0,02.

### Ethical considerations

Ethical approval was obtained from Ethic Committee of University of Seville. Every questionnaire had a brief explanation about aims of research and asked for informed consent. Those students who filled it in consented to participate in the study. All questionnaires were collected by an investigator from the research team. Confidentiality was guaranteed using anonymous questionnaires and participants were informed about the right to not answer.

### Statistical methods

Data were analyzed using Statistical Package for Social Science (SPSS) version 22.0. Descriptive analysis was used to describe participants’ sociodemographic characteristics, sexual behaviors, knowledge and use of contraception. Student’s t-test was used to compare means between groups for normally distributed continuous variables and U Mann Whitney was used for skewed continuous data. Quantitative variables were compared by Pearson chi-square test. The chi square test was used to determine the association between categorical variables such as gender, grouped age, knowledge area, and sexual behavior. A *P*-value of 5% (two-tailed) was considered statistically significant.

## Results

A total of 566 students participated with a response rate of 98%. 12 students (2%) did not return the questionnaire filled in. As is shown in Table 1, most participants (94.8%) were aged under 25 years-old, 47.3% (267) were men and 52.6% (297) women. Among the different religious affiliations, catholic (58.7%) was the most prominent. Among responding students, 55.7% lived with family during the course; 508 (92.5%) most respondents referred to be heterosexuals.

**Table 1:** Sociodemographic characteristics of study participants (*n* = 566)

	Number	Percent
Age^a^		
< 20	151	27.1
21–24	377	67.7
> 25	29	5.2
Sex		
Women	297	52.6
Men	267	47.3
Religion^b^		
Catholic	307	58.9
Muslim	7	1.2
Atheism/Agnosticism	215	38.7
Others	37	1.2
Residence^b^		
Parents	314	55.7
Shared flat	195	34.5
University residence	23	4.1
With partner	31	5.5
Others	3	0.2
Sexual identity^c^		
Heterosexual	508	92.5
Homosexual	23	4.2
Bisexual	18	3.3
Previous information^b^		
Yes	373	65.3
No	193	34.7
Education^d^		
From School	264	90.4
Family	12	4.1
Internet	2	0.7
Health Centre/Health providers	14	4.8

^a^ *n* = 557; ^b^ *n* = 566, ^c^ *n* = 549, ^d^ *n* = 292

65.3% received sexual and contraceptive education. Main sources of information about sexuality are school in 90.4%; healthcare professionals (4.8%), friends/family (4.1%) and Internet (0.7%).

### Sexual behavior

93.3% of participants had already had sexual intercourse. Their sentimental situation was single (not involved in a sentimental relationship) of 44.5%. 55.1% (311) affirmed to have a partner; for under a year 23.1%, between 1 and 2 years 18.4% and over 2 years, 45.2%. Age of first intercourse was M (SD) = 17.03 (6.43) years-old and 78.4% had their sexual relationship with their boyfriend/girlfriend. 95.5% of participants had had some kind of sexual relationship.

91.7% had only had a sexual partner last year, 6.2% more than one, and 2.1% more than two. Frequency of sexual relations was 1–2 times in a week for 50.1%, 18.9% 1–2 in a month, 9.2% 1–2 in a year, and 21.8% more than twice in a week.

41.7% had never had sexual relations under alcohol effect, 57.2% sometimes, and 1.1% always drank alcohol in the context of sex. However, under drugs effects, never 82% and 16.7% sometimes, and always consumed drugs and had sex 1.3%. 52.7% always enjoyed their sexual relations, 40.7% sometimes, and never 6.6%. A 9% referred to having been forced to have sex.

### Contraceptive behavior

86.9% used a contraceptive method during their first sexual relation, the male condom being the most used (90.6%). An 8.2% used coitus interruptus.

Currently, 8.1% use coitus interruptus as a method, 64% condom and 23.4% hormonal pills.

A total of 32.6% had used emergency contraception: 21.3% used a post coital pill, at least, once; and 11.4% more than once. A 2.6% confirmed having had a legal abortion.

### Knowledge and attitude about contraceptives and TSI

The participants’ answers about their knowledge on contraceptives and TSIs are shown in Table 2. We can observe that lower scores are related with knowledge about TSI. The median of the global score was 7.11 points, minimum 2 points and maximum 8 points and SD = 1.20. 45.1% had full right answers and 26.7% had 7 right responses. Item 9 was the most failed, right answers were between 78.9 and 97.7%.

**Table 2:** Scores of scales about contraceptive use and sexual behavior

Items	True N (%)	False N (%)	% correct
Coitus interruptus is a very safe contraceptive.	16 (2.9)	538 (97.1)	97.1
if condom is washed carefully, it is possible to reuse it several times.	13 (2.3)	541 (97.7)	97,7
To use hormonal pills, you have to appoint a general practitioner at primary health Centre	459 (84.5)	83 (15.3)	84,5
Having sex during menstruation is safe to prevent pregnancies	43 (7.8)	507 (92.2)	92,2
Hormonal pills are effective if you take it before sex	77 (14.2)	464 (85.8)	85,8
Hormonal pills protect against STI	42 (7.7)	507 (92.3)	92,3
The only method that protects you against STI is condom	439 (80)	110 (20)	80,0
All women active sexually has to perform a Papanicolau annually	419 (78.9)	112 (21.1)	78,9
STI are common in our society	75 (13.9)	465 (86.1)	13,9

Participants who had received sexual and contraceptive education showed more knowledge M(SD) = 7.28 (1.04) vs 6.82 (1.41), *p* < 0.001. On the other hand, respondents that were in a relationship had more knowledge, 7.31 (1.02) vs 6.87 (1.35), *p* < 0.001. Those women who had had a legal abortion obtained a lower score in the scale 6.43 (1.95) vs 7.13 (1.18), *p* = 0.034.

We did not find differences about knowledge on contraceptives and STIs by age, *p* = 0.056.

Level of knowledge is less in young people who use coitus interruptus or none as contraceptive method, *p* < 0.001. However, those that use a hormonal method or double method showed better scores.

Finally, we observed differences by frequency of sex since young people who had sex more frequently showed more knowledge about contraceptives and STI, *p* < 0.001.

### Comparison by gender, age and studies

As can be seen in Table 3 there are many differences among participants by gender.

**Table 3:** Distribution of contraceptive use and sexual behavior by sex

	Women *N* = 297	Men *N* = 267	*p value* ^*a*^
	n (%)	n (%)	
First sexual intercourse with a boyfriend	236 (79.46)	158 (59.2)	*P* < 0.001
Contraceptive use in first intercourse	236 (91.1)	195 (73.03)	*P* = 0.004
Current contraceptives use	255 (85.8)	234 (87.6)	*p* > 0.05
Currently in sexual relationship	183 (61.6)	127 (47.6)	*P* = 0.002
Ever had sexual intercourse	21 (7.07)	22 (8.2)	*p* > 0.05
Always satisfactory sex	118 (39.7)	144 (53.93)	*P* = 0.008
Ever used emergency contraception	172 (57.91)	182 (68.16)	*P* = 0.07
Had unsafe sex in past year	23 (7.74)	42 (15.7)	*P* = 0.01
Number of sexual partners in the past year, *M (SD)*	1.07 (0.28)	3.47(2.04)	*P* = 0.005
Frequency of sex			
1–2 in a week	21 (7.07)	88 (32.95)	
1–2 in a month	130 (43.77)	50 (18.72)	*P* = 0.029
1–2 in a year	48 (16.16)	34 (12.73)	
More than twice a week	14 (4.71)	56 (20.97)	
Had sex under drugs effect	31 (10.43)	68 (25.46)	*P* = 0.000
Had sex under alcohol effect	164 (55.21)	157 (58.8)	*p* > 0.05
Age at first sex, *M (SD)*	17.48 (9.13)	16.63 (1.97)	*p* > 0.05
Current contraceptive method			*P* = 0.01
Coitus interruptus	19 (6.39)	21 (7.86)	
Condom	151 (50.84)	167 (62.54)	
Hormonal pills	78 (26.26)	37 (13.85)	
Others	7 (2.35)	9 (3.37)	
Had an abortion	7 (2.35)	7 (2.62)	*p* > 0.05
Sources of information			
School	145 (48.82)	119 (44.56)	
Family	6 (2.02)	6 (2.24)	*P* = 0.001
Internet	0 (0)	2 (0.74)	
Heath providers	14 (4.71)	0 (0)	
Information about STIs and contraceptives right answers	n (%)	n (%)	*p value* ^*a*^
Coitus interruptus is a very safe contraceptive.	286 (96.29)	251 (94)	*P* = 0.213
if condom is washed carefully, it is possible to reuse it several times.	287 (96.63)	253 (94.75)	*P* = 0.110
To use hormonal pills, you have to appoint a general practitioner at primary health centre	262 (88.21)	196 (73.4)	*P* = 0.000
Having sex during menstruation is safe to prevent pregnancies	275 (92.59)	231 (86.51)	*P* = 0.062
Hormonal pills are effective if you take it before sex	268 (90.23)	195 (73.03)	*P* = 0.000
Hormonal pills protect against STI	275 (92.59)	231 (86.51)	*P* = 0.043
The only method that protects you against STI is condom	246 (82.82)	192 (71.91)	*P* = 0.004
All women active sexually has to perform a Papanicolau annually.	242 (81.48)	176 (65.91)	*P* = 0.000
STI are common in our society	32 (10.77)	43 (16.10)	*P* = 0.056

^a^Significance tested using *F* test for continuous variables and 2 test for categorical variables

There are more women that had a partner than men (*p* = 0.003) and their attitudes and experiences on sex are healthier. Females showed more knowledge about management of hormonal contraceptives and medical controls.

Students older than 25 years old were more often in a romantic relationship (*p* = 0.003). Aged group between 20 and 25 years had long relationships, over 2 years (*p* < 0.001). Related to contraceptives, the most used in aged group 20 is the condom and 20–25 years, hormonal pills (*p* < 0.001). Age is not related with use of emergency contraception (*p* = 0.098), neither to perform a legal abortion (*p* = 0.356).

Number of sexual partners in last 12 months is reduced with age, thus frequency of sex (*p* = 0.02). Use of alcohol with sex is more often at 20–25 years (*p* < 0.001) and drugs at over 25 years (*p* < 0.001).

Age group between 20 and 25 are most satisfied sexually (*p* < 0.001) if compared with other age groups.

Participants that studied a degree in Health Sciences had attended some kind of formation on sexuality and contraception, mainly, at school, in comparison with other fields (*p* < 0.000).

Use of a contraceptive in the first sexual intercourse showed differences depending on the degree, since students of a degree in Engineering or Architecture did not use any method if compared with other fields (*p* = 0.04). Otherwise, they most frequently used an inconsistent method such as coitus interruptus (*p* = 0.03). When we compared knowledge and sexual practices among students of different areas of knowledge, we observed that students enrolled in some degree of health sciences showed healthier behavior than other areas *p* < 0.001.

## Discussion

This study evaluated the knowledge, behaviors, perceptions and use of contraceptives and sexuality among students at University of Seville. Our results showed that the knowledge and sexual practices are suboptimal so it is necessary to continue education at university levels.

Our data described that most students were sexually active at university period. They had their first sexual intercourse at the age of 17, slightly inferior to national statistics that referred the age at 18.2 [24] but higher than the age found by Rodríguez- Carrión et al. that stated at 14 years old [23]. In 2016, Contraception Spanish Society (CSS) published results from a National Survey with 2.200 participants aged 15–49. The main findings of this report are in line with ours about contraceptive pattern on the first sexual intercourse, 2 out of 10 adolescents do not use a contraceptive for their first sexual intercourse, where this most commonly occurs in males [24]. This is based on the assumption that men adopt a riskier attitude than female students. Previous studies already demonstrated that women had a better knowledge and more preventive attitude to risk-taking [25]. This is in line with our findings, women are shown to have more information and adopt healthier behaviors related to sexual relations and use of contraceptives. In general, females showed a greater knowledge and had a safer attitude during first intercourse using a contraceptive. They have more stable sentimental relations while males have more sexual partners in a year contributing to a higher exposure to risks. Other studies reported that males had higher rates of frequency of sex [26] and lower rates of contraception [27]. Besides, females showed more awareness about medical controls during this period of life as shown in the scores in the scale.

Consumption of alcohol in young people is more frequent every day, independently of sex. As women and men used alcohol for fun, because of this, it is very usual that both groups have sex under alcohol effects [28]. But not only alcohol, sometimes under drugs effects as well. Among the sexually active students, 56.8% had a sexual intercourse under alcohol effects and 17.9% were engaging in unsafe sex under drugs presence. The reported prevalence of unsafe sexual was higher in males, although just descriptively. On the other hand, when we compared between females and males having sex under drugs effects, we found that males over 25 were most common. A riskier behavior is reported when they had sex associated with alcohol and drugs [29]. Similarly, Brown et al. observed that alcohol use leads to greater risk-taking behavior including non-use of contraceptive [30]. Rodríguez- Carrión et al. found that adolescents consumed alcohol or drugs before their first sexual intercourse [23]. However, in a multi-country study carried out in 22 countries, it was found that alcohol use in the context of sex did not have a negative effect on contraceptive use [31] and this result is in line with our findings.

In our study, nearly 93.3% of the students were sexually active in the past 12 months. Only 7.8% had never had sex, very similar to 8.3% of the recent national survey of CSS.

The most used contraceptive is the condom among under 20 years and contraceptive pill in 20–25 years group. This finding is in line with national data and reported in other studies [32, 33].

A rate of 32.6% used emergency contraception. We did not ask about motivation to use it, but usually it is used in the case of unprotected sexual intercourse or failure of a regular contraceptive method, so young people should be made aware of this. A recent study with Scandinavian women showed lower rates of use, under 10%, so we should include this in future programs to make students aware that this is not a contraceptive [34].

Most people aged between 20 and 24 years showed reasonable knowledge about contraception and STI, although these results are improved with age using a consistent method and taking less risks, specifically for females. We observed a general banalization of STI, young people think that are not frequent in our society, despite statistics showing they have been increasing in the last years [5, 6]. This is a topic that should be covered deeply in sexual education for young people at university.

Nearly 65.3% of the respondents in this survey reported having received school- based sexual education before enrolling at University. Students with higher sexual and reproductive health knowledge were those that attended some kind of intervention during High School and used a consistent contraceptive method. As in other studies, young people that attended a school based sex program were more likely to use an effective contraceptive method after 12 months intervention [33, 35].

Not only knowledge protects against STIs. Lopez et al. [33] found that students that attended sexual education programs used condom and some hormonal method more frequently. It is necessary to continue young people’s awareness at university as well. Some of them received information at school and despite that, adopt risk conducts. A continuing emphasis on giving information about sexuality and contraceptives is crucial for the promotion of a healthy sex life including the correct use of contraceptives and healthy sexual behaviors emphasizing on STI and its current prevalence.

The most common sources of information regarding sexual health and contraception were friends, television and health providers. Similar findings were found in another study with 1900 young people, females were more likely to obtain contraceptive information from health care professionals, whereas males were more likely to report friends, partners, internet and television/radio [36]. However, we do not assess whether knowledge and/or information had a positive impact on behaviors. We observed that those that were more informed had healthier lifestyle, but we cannot state that this behavior is due to previous education. Studies carried out in other countries that evaluated sources of information about contraceptives found that a high knowledge from sources was not enough to result in actual use [37].

In Spain where this study was conducted, there is still a lack of an evaluation system of health education programs. A study carried out by Lima et al. and Morales et al. concluded that adolescents and young people who received sexual education had similar conducts to those that did not [2, 20]. More studies that assess the impact of these programs on young conducts are needed. Regarding this, with the results of this survey we propose to continue teaching sexual education at university since students continue showing risk behaviors and lack of knowledge.

### Strengths and limitations of the study

We may consider some limitations in this research. First, due to nature of questions, information bias could be provided by students and this may affect reliability of the results due to social desirability. Second, we cannot establish a cause and effect relationship due to this being a cross-sectional design. This study was conducted in one university, the results are therefore not necessarily generalizable to other universities in Spain.

A small proportion of students declined to answer, but even so, we do not know their reasons to do so.

As a strength, this study provides information about university students and their sexual and contraceptive attitudes to design education programs on sexuality and reproductive issues.

In the future, it would be interesting to explore what strategies are successful in reducing risk behaviors.

## Conclusions

This study showed that there are factors that contribute to having a healthier behavior on sexuality and contraception: older students, women and students of health sciences career showed a healthier behavior in sexuality and a higher level of knowledge. Those who had received some education about sexuality and contraception showed greater knowledge and less risky behavior. However, we observed this is not enough and should be improved by education, also at University stages, in order to avoid students taking risks. Specially, education must be focused on sexually transmitted infections and the correct use of contraceptives.

It is necessary to continue programing reproductive health programs at university to improve sexual and reproductive attitudes among university students and assess the impact that they have on their lifestyles.
